# Antivenom Effects of 1,2,3-Triazoles against *Bothrops jararaca* and *Lachesis muta* Snakes

**DOI:** 10.1155/2013/294289

**Published:** 2013-04-22

**Authors:** Thaisa F. S. Domingos, Laura de A. Moura, Carla Carvalho, Vinícius R. Campos, Alessandro K. Jordão, Anna C. Cunha, Vitor F. Ferreira, Maria Cecília B. V. de Souza, Eladio F. Sanchez, André L. Fuly

**Affiliations:** ^1^Programa de Pós-Graduação em Biologia Marinha, Instituto de Biologia, Universidade Federal Fluminense, Niterói, RJ, Brazil; ^2^Programa de Pós-Graduação em Biologia das Interações, Instituto de Biologia, Universidade Federal Fluminense, Niterói, RJ, Brazil; ^3^Departamento de Biologia Celular e Molecular, Instituto de Biologia, Universidade Federal Fluminense, Outeiro de São João Batista, s/n, 3 Andar, Sala 310, 24020-141 Niterói, RJ, Brazil; ^4^Departamento de Química Orgânica, Programa de Pós-Graduação em Química, Universidade Federal Fluminense, Niterói, RJ, Brazil; ^5^Fundação Ezequiel Dias, Centro de Pesquisa e Desenvolvimento, Belo Horizonte, MG, Brazil

## Abstract

Snake venoms are complex mixtures of proteins of both enzymes and nonenzymes, which are responsible for producing several biological effects. Human envenomation by snake bites particularly those of the viperid family induces a complex pathophysiological picture characterized by spectacular changes in hemostasis and frequently hemorrhage is also seen. The present work reports the ability of six of a series of 1,2,3-triazole derivatives to inhibit some pharmacological effects caused by the venoms of *Bothrops jararaca* and *Lachesis muta*. *In vitro* assays showed that these compounds were impaired in a concentration-dependent manner, the fibrinogen or plasma clotting, hemolysis, and proteolysis produced by both venoms. Moreover, these compounds inhibited biological effects *in vivo * as well. Mice treated with these compounds were fully protected from hemorrhagic lesions caused by such venoms. But, only the *B. jararaca* edema-inducing activity was neutralized by the triazoles. So the inhibitory effect of triazoles derivatives against some *in vitro* and *in vivo* biological assays of snake venoms points to promising aspects that may indicate them as molecular models to improve the production of effective antivenom or to complement antivenom neutralization, especially the local pathological effects, which are partially neutralized by antivenoms.

## 1. Introduction

Snake venoms are complex mixtures of proteins including enzymes (metalloproteinases, serine proteinases, phospholipases A_2_, and L-amino acid oxidase) and proteins without enzymatic activity, such as disintegrins, C-type lectins, cysteine-rich secretory proteins (CRISP) toxins, natriuretic peptides, and myotoxins. The venomous pit vipers *Bothrops jararaca* and *Lachesis muta* are responsible for accidents involving humans in several regions of South America. While *B. jararaca* is found in southern Brazil, Paraguay, and northern Argentina, *L. muta* is distributed in the equatorial forests east of the Andes, ranging from eastern Ecuador, Colombia, Peru, northern Bolivia and eastern and northern Venezuela, to Guyana, French Guyana, Surinam, and northern Brazil. Within their range, they are often abundant and are important cause of snakebites [1]. Envenoming by these snakes is mainly characterized by systemic (generalized bleeding, coagulopathy, renal failure and shock) and local effects (hemorrhage, edema, and necrosis) [2–4]. As reported elsewhere, snake bites constitute a public health problem in Latin America and in other tropical and subtropical countries, in which they are considered as a neglected health issue, according to the World Health Organization (WHO) [5]. In South America, *B. jararaca* induces a higher incidence of bites (95%) than *L. muta* (circa 2%); however, *L. muta* bites usually lead to more severe envenoming symptoms and its lethality incidence is three times higher than *B. jararaca *[5]. Nowadays, the parenteral administration of animal-derived antivenom is the only specific treatment for envenoming by snakebites. In Brazil, the intravenous administration of either *Bothrops* polyvalent antivenom is used to treat the envenoming cases caused by *Bothrops* bites or the polyvalent bothropic-lachetic serum for *L. muta* and *Bothrops* (*B. atrox*) snake bites in the Amazonian regions. As stated above, moderate to severe envenomings inflicted by *Bothrops *and *Lachesis* snakes are characterized by a complex series of local and systemic alterations such as hemorrhage, myonecrosis, coagulopathy, cardiovascular shock, renal failure, and eventually death [6]. As reported by other authors, despite of being safe, high doses of antivenoms sometimes used in Brazil to treat patients with proven or suspected *Bothrops*/*Lachesis* envenoming may contribute to early anaphylactic and late (serum sickness) type reactions [7]. Thus, the production of antivenoms of adequate quality presents a considerable challenge. Moreover, the prices of antivenoms have increased and some countries have stopped their manufacture [5]. Some antivenoms efficiently neutralize the systemic toxic effects of the venom; however, the local effects are not blocked and this situation can lead to amputation or disability [8].

Because of such problems, alternative treatments have been sought and some of them have involved the search for new molecules able to neutralize systemic and local effects of venoms. Extracts from plants and other natural sources (as those from marine organisms) have been tested for their ability to neutralize a variety of biological and toxic effects of snake venoms. Various pharmacologically active molecules have been identified, and many effects have already been listed for them [9–12], including their antivenom ability [13, 14]. Nowadays, many new bioprospecting approaches are being investigated. However, in connection with this, it should be noted that as yet the biological effects of molecules derived from organic synthesis have not been well explored. Literature has described 1,2,3-triazole compound as an important class of five-member nitrogen heterocyclic system which exhibits different pharmacological profiles, such as antiplatelet activity [15], anticlotting [16], antiviral [17], trypanocidal [18], antimicrobial [19], and/or their use in treating schizophrenia [20] and leishmaniasis [21]. Two general methods are available for the construction of 1,2,3-triazole rings: Huisgen 1,3-dipolar cycloaddition reactions [22], in particular the copper(I)-catalyzed cycloaddition [23], and the intramolecular 1,5-electrocyclization of *β*-substituted-*α*-diazocarbonyl compounds [24]. Our previous studies have indicated that six new synthetic 1,2,3-triazole compounds (1-arylsulfonylamino-5-methyl-1*H*-[1,2, 3]-triazole-4-carboxylic acid ethyl esters) inhibited the hemolysis induced by *L. muta* venom [25]. In fact, such derivatives displayed a wide range of pharmacological activities [15–25].

The aim of this work was to evaluate the ability of these six 1,2,3-triazole derivatives based on *N*′-[(4′-bromophenyl)methylene)]-1-(*p*-chlorophenyl)-1*H*-[1,2, 3]-triazole-4-carbohydrazide against *in vivo* and *in vitro* activities of *Bothrops jararaca* and *Lachesis muta* venoms.

## 2. Material

### 2.1. Venom and Material

*Bothrops jararaca*, *Lachesis muta* lyophilized venoms, and anti-Lachesis or anti-Bothropic antivenom were provided from Fundação Ezequiel Dias, Belo Horizonte, MG, Brazil, and stored at −20°C until assays. Dimethylsufoxide (DMSO), bovine fibrinogen, and azocasein were obtained from Sigma Chemical Co. All other reagents were of the best grade available.

### 2.2. Synthetic Derivatives

**Figure 1:**
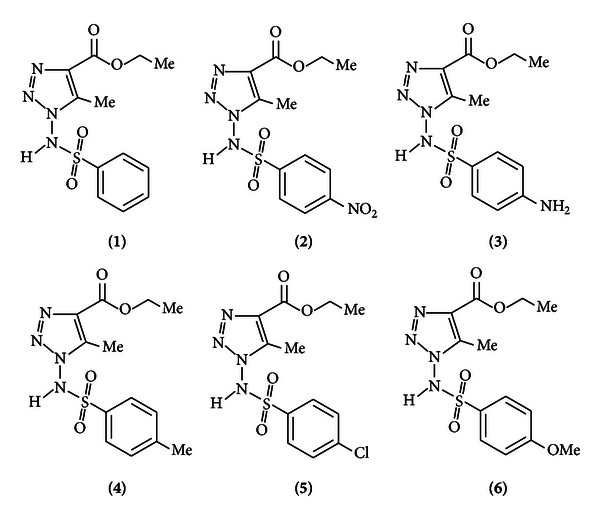
Chemical structures of the six 1,2,3-triazoles derivatives *N *′-[(4′-bromophenyl)methylene)]-1-(*p*-chlorophenyl)-1*H*-[1,2, 3]-triazole-4-carbohydrazide. The six derivatives were designed as numbers, as shown in the parenthesis after each derivative.

The six 1-arylsulfonylamino-5-methyl-1*H*-[1,2, 3]-triazole-4-carboxylic acid ethyl esters derivatives were synthesized according to our previous report [17] and their chemical structures are shown in Figure 1. These compounds were dissolved in dimethylsufoxide (DMSO) and stored at 4°C, until required.

### 2.3. Animals

BALB/c mice (18–20 g) were obtained from the Núcleo de Animais de Laboratório (NAL) of the Federal Fluminense University. The animals were housed under controlled conditions of temperature (24 ± 1°C) and light. Experiments were approved by the UFF Institutional Committee for Ethics in Animal Experimentation (protocol number 297) that were in accordance with the guidelines of the Brazilian Committee for Animal Experimentation (COBEA) and international laws and policies. 

## 3. Methods

### 3.1. Inhibition of Indirect Hemolysis

The degree of hemolysis caused by the venoms of *L. muta *and* B. jararaca* was determined by the indirect hemolytic test using human erythrocytes and hen's egg yolk emulsion as substrate [26]. The amount of *L. muta *and* B. jararaca* venom (*μ*g/mL) that produced 100% hemolysis was denoted as minimum indirect hemolytic dose (MIHD). Inhibitory experiments were performed by incubating triazole derivatives with one MIHD for 30 minutes at room temperature, and then, hemolytic activity was evaluated. Control experiments were done by incubating venoms with DMSO or saline solution.

### 3.2. Anticlotting Activity

The clotting activity of *L. muta* and* B. jararaca* venoms was determined using a digital Amelung coagulometer, model KC4A (Labcon, Germany). Different concentrations of *L. muta* (10 *μ*g/mL) and* B. jararaca* (40 *μ*g/mL) venom were mixed with bovine fibrinogen solution (2 mg/mL) or with human plasma, and the amount of venom that clotted either fibrinogen or plasma in 60 seconds was denoted as minimum coagulant dose (MCD). To evaluate their inhibitory effect, the triazole derivatives were incubated for 30 minutes at room temperature with one MCD of venoms, and then the mixture was added to fibrinogen or plasma and clotting time recorded. Control experiments were performed in parallel by adding DMSO or saline incubated with venoms, instead of the triazoles.

### 3.3. Antiproteolytic Activity

Proteolytic activity of *L. muta* and* B. jararaca* venoms was determined using azocasein as substrate (0.2% w/v, in 20 mM Tris-HCl, 8 mM CaCl_2_, pH 8.8), with minor modification [13, 27]. An effective concentration (EC) was defined as the amount of venom (*μ*g/mL) able to produce a variation at A 420 nm of about 0.2. Triazole derivatives were incubated with one EC of venom for 30 minutes at room temperature and then proteolysis was measured. Control experiments were done by incubating venoms with DMSO or saline solution.

### 3.4. Antihemorrhagic Activity

Hemorrhagic lesions produced by *L. muta* and* B. jararaca* venoms were quantified using a procedure described by Kondo et al. [28], with modifications. Briefly, samples were injected intradermally (i.d.) into the abdominal skin of mice. Two hours later, the animals were euthanized by decapitation, abdominal skin removed, stretched, and inspected for visual changes in the internal aspect in order to localize hemorrhagic spots. Hemorrhage was quantified as the minimum hemorrhagic dose (MHD), defined as the amount of venom (mg/kg) able to produce a hemorrhagic halo of 10 mm [29]. The inhibitory effect of triazole derivatives was investigated by incubating compounds with two MHD of *L. muta* or* B. jararaca* venom for 30 minutes at room temperature and then the mixture was injected into mice and hemorrhage was measured. Hemorrhagic activity was expressed as the mean diameter (in millimeter) of the hemorrhagic halo induced by venoms in the absence and presence of the triazole derivatives. Negative control experiments were performed by injecting DMSO or saline solution.

### 3.5. Antiedematogenic Activity

Edema-inducing activity of *L. muta* and* B. jararaca* venoms was determined according to Yamakawa et al. [30], with modifications. Groups of five mice were injected subcutaneously (s.c) in the right foot pad with 50 *μ*L of venom, whereas the left food pad received 50 *μ*L of saline. One hour after injection, edema was evaluated and expressed as the percentage of increase in the weight of the right foot pad compared to the left one. The inhibitory effect of triazole derivatives was investigated by incubating compounds with *L. muta* or* B. jararaca* venom for 30 minutes at room temperature and then the mixture was injected into mice (right foot pad) and edema was measured.

### 3.6. Statistical Analysis

Results are expressed as means ± SEM obtained with the indicated number of animals or experiments performed. The statistical significance of differences among experimental groups was evaluated using the Student's *t*-test. A *P* value of ≤ 0.05 was considered significant.

## 4. Results and Discussion

The development of effective, safe, cheaper, and more accessible antivenoms deserves attention, since snake bites may cause severe disabilities and kill thousands of people as well. A growing number of studies have been focused on the search for inhibitors of snake venoms from a variety of sources, be they natural or synthetic [31, 32]. Suramin [33–35] and benzoyl phenyl benzoate [36] are synthetic molecules able to inhibit myotoxicity, clotting, and phospholipase A_2_ and hyaluronidase activities of snake venoms from different families. Lactone analogs were synthesized and inhibited myotoxicity, and edema-inducing and enzymatic activities induced by a phospholipase A_2_ isolated from *B. jararacussu* either [37]. On the other hand, marine bioactive principles have also attracted attention because of their wide spread pharmacological actions [38].

**Figure 2:**
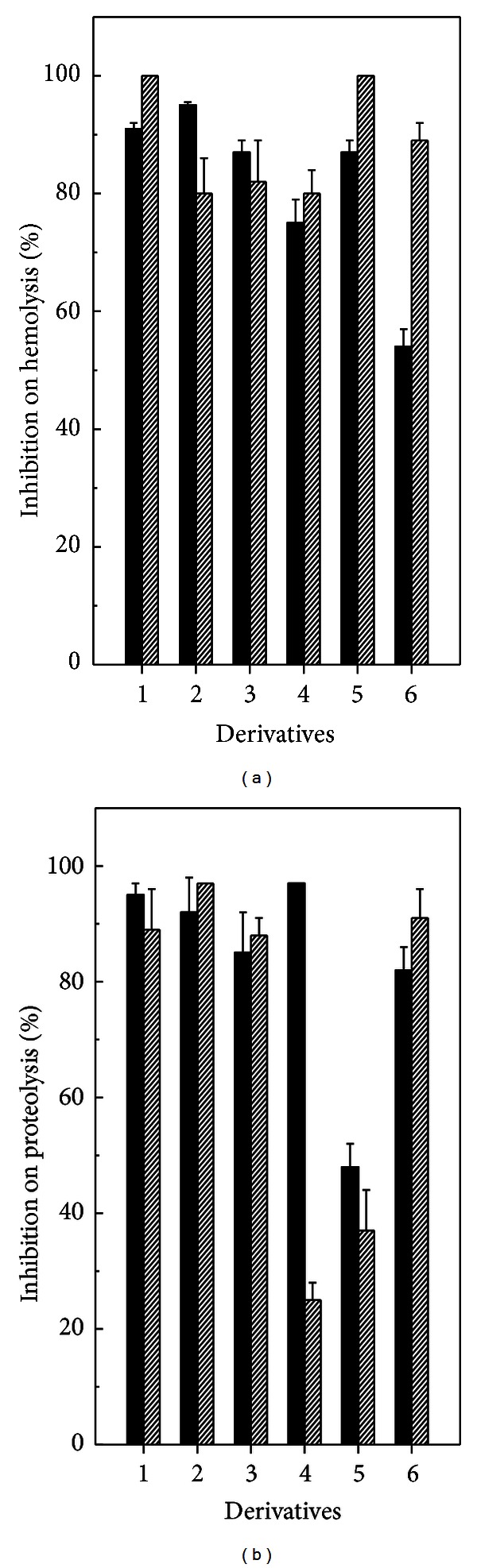
Effect of derivatives on hemolysis and proteolysis. Derivatives **1**–**6** (45 *μ*M) were incubated with *B. jararaca* (dark columns) or with *L. muta* (dashed columns) for 30 minutes at room temperature, and then hemolytic (a) and proteolytic (b) activities were performed. Data are expressed as mean ± SEM of individual experiments (*n* = 3).

In this work, it was evaluated the ability of six 1-arylsulfonylamino-5-methyl-1*H*-[1,2, 3]-triazole-4-carboxylic acid ethyl esters to neutralize some *in vitro* (hemolysis, clotting, and proteolysis) and *in vivo* (hemorrhage, and edema-inducing) activities caused by *B. jararaca* and *L. muta *venoms, since previous results indicated that these six derivatives inhibited hemolysis induced by *L. muta* venom, but with different potencies [25]. For this reason, it was thought it would be worthwhile to investigate the actions of such derivatives upon other important biological activities related to snake bites, as proteolysis, clotting, hemolysis, hemorrhage, and edema. It was showed that these compounds inhibited the hemolysis caused by *B. jararaca* (50 *μ*g/mL) and *L. muta* (15 *μ*g/mL) venom (Figure 2(a)). The inhibitory percentage of the derivatives was above 50% against both venoms. However, a slight difference on the inhibitory profile was observed for derivative **6**, where a 50% and 90% inhibition on hemolysis was achieved for *B. jararaca* and *L. muta* venom, respectively. Neither derivatives nor DMSO led erythrocytes to hemolysis, nor did DMSO interfere in the degree of hemolysis caused by venoms.

Envenomation by these snakebites produces severe hemorrhage due to the high content of zinc-dependent metalloprotease or serine protease that digest protein components of the extracellular matrix or consume blood clotting factors [39]. *B. jararaca* and *L. muta* venom hydrolyzed azocasein in a concentration-dependent manner with an EC of 20 *μ*g/mL and 6 *μ*g/mL, respectively (data not shown). The derivatives inhibited proteolysis induced by *B. jararaca* or *L. muta* (Figure 2(b)). The derivatives **1**, **2**, **3**, and **6** inhibited proteolysis induced by both venoms up to 80% and the derivative **5** inhibited such an activity below 50%. A marked difference on inhibitory profile of derivatives was observed for derivative **4**, in which it inhibited 97% and 25% the proteolysis induced by *B. jararaca* or *L. muta* venom, respectively (Figure 2(b)).

**Figure 3:**
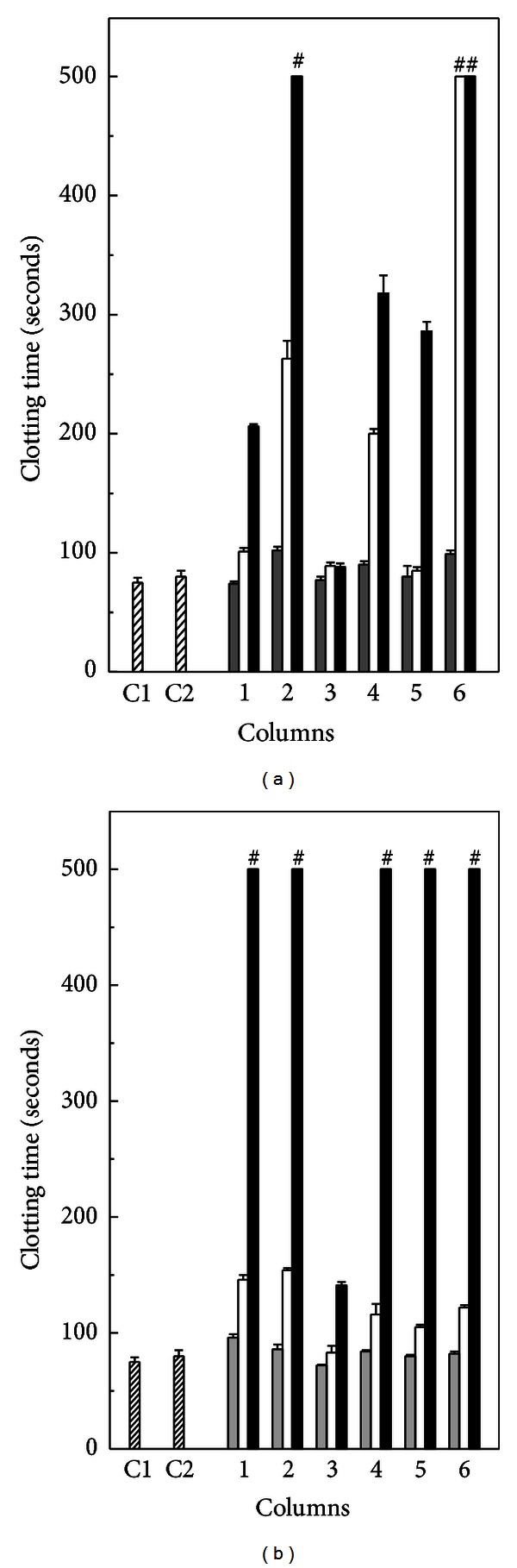
Effect of derivatives on fibrinogen clotting. Twenty three *μ*M derivatives (gray columns), 46 *μ*M (white columns), or 94 *μ*M (black columns) were incubated with 40 *μ*g/mL *B. jararaca* (a) or with 10 *μ*g/mL *L. muta* (b) for 30 min at room temperature. Then, mixture was added to fibrinogen (2 mg/mL) and clotting time was recorded. Venoms were incubated with saline (C1); 1% v/v DMSO (C2); derivative **1** (column 1); derivative **2** (column 2); derivative **3** (column 3); derivative **4** (column 4); derivative **5** (column 5); and with derivative **6** (column 6). # means that fibrinogen did not clot until 600 seconds of observation. Data are expressed as mean ± SEM of individual experiments (*n* = 4).

As seen in Figure 3, derivatives **1**, **2**, **4**, **5** and **6**, but not the derivative **3** inhibited in a concentration-dependent manner (23–94 *μ*M), the clotting of fibrinogen induced by the venoms of *B. jararaca* (40 *μ*g/mL) or *L. muta* (10 *μ*g/mL). It seemed that derivatives inhibited more efficiently *L. muta*-inducing clotting than *B. jararaca*. At the highest concentration (94 *μ*M), the derivatives **1**, **2**, **3**, **5** and **6** prevented *L. muta* clotting (Figure 3(b)), while the derivatives **2** and **6** prevented the *B. jararaca* once (Figure 3(b)). At concentrations up to 200 *μ*M, all the 1,2,3-triazole derivatives effectively prevented fibrinogen clotting caused by both venoms, but at concentrations below 10 *μ*M, none of these compounds prevented clotting. However, when derivatives (10 *μ*M) were put all together and incubated with either *B. jararaca* or *L. muta* venom, the clotting time was delayed two-fold. It was noticed that, if derivative **2** or **6** was removed from mixture, inhibitory effect upon clotting was not observed. Moreover, the derivatives also prevented clotting induced by venoms when plasma was used. Neither DMSO (1% v/v, final concentration) nor saline solution interfered with coagulation processes.

**Figure 4:**
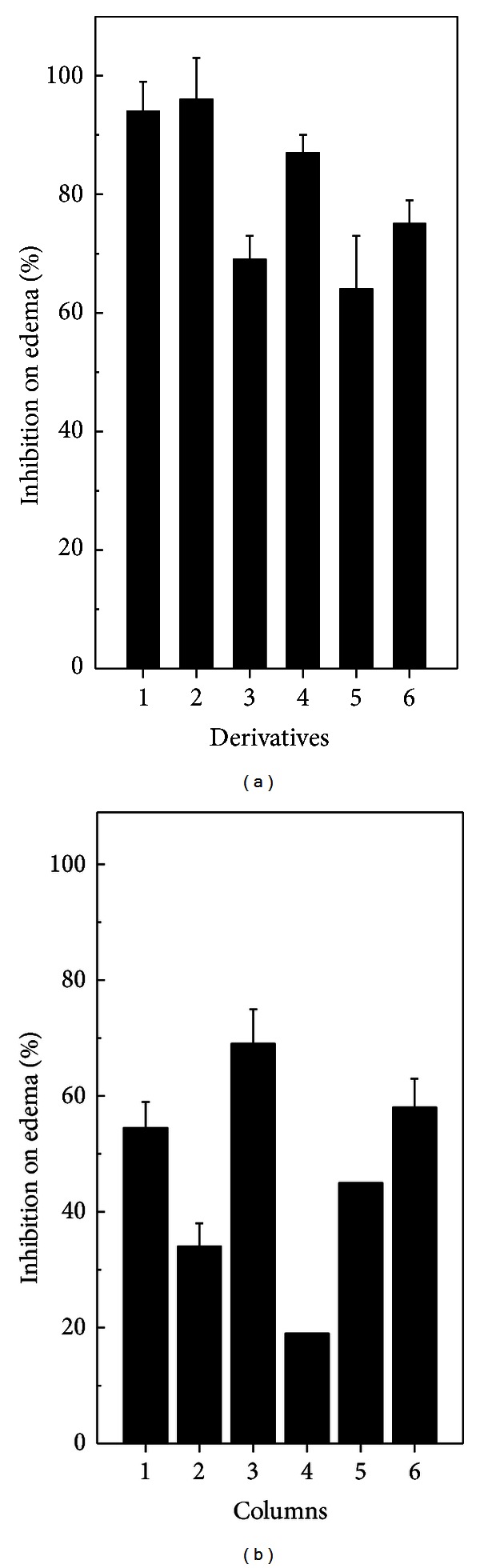
Effect of derivatives on edema-inducing activity. The derivatives (90 *μ*M) were incubated with 5 mg/Kg of *B. jararaca* (a) or with 8 mg/Kg *L. muta* (b) for 30 minutes at room temperature, and then edema-inducing activity was performed. Columns are derivative **1** plus venom (1); derivative **2** plus venom (2); derivative **3** plus venom (3); derivative **4** plus venom (4); derivative **5** plus venom (5) and derivative **6** plus venom (6). Data are expressed as mean ± SEM of individual experiments (*n* = 4).

Intradermal injection of *B. jararaca* (12 mg/Kg) or *L. muta *(20 mg/Kg) venom produced a hemorrhage halo of 20 mm in mice. Such a halo represents two MHD of venoms. When each venom was mixed with derivatives (90 *μ*M) and then injected into mice, a complete protection from hemorrhage was seen (data not shown). In contrast, previous results showed that antilaquetic serum did not inhibit hemorrhage induced by *L. muta* venom [13]. Injection of DMSO, derivatives or saline solution did not produce hemorrhage. Edema-inducing is another important effect that follows snake bite [3, 40].  Figure 4 shows that edema induced by 5 mg/Kg *B. jararaca* (Figure 4(a)) or 8 mg/Kg *L. muta* (Figure 4(b)) was significantly reduced by the derivatives (90 *μ*M). The triazole derivatives **1**, **2**, and **4** inhibited above 80% the edema induced by *B. jararaca*, whereas derivatives **3**, **5**, and **6** inhibited around 70% (Figure 4(a)). As seen, all derivatives inhibited less *L. muta*-induced edematogenic activity (Figure 4(b)).

In conclusion, 1-arylsulfonylamino-5-methyl-1*H*-[1,2, 3]-triazole-4-carboxylic acid ethyl esters derivatives may be useful as prototypes for designing new molecules to improve the current treatment used for *B. jararaca* and *L. muta* snake bites. The inhibitory potency of these derivatives may vary or may be enhanced when they were put all together, probably acting synergistically. Thus, a lower concentration of them would be needed to reach a complete neutralization of the biological effects caused by *B. jararaca* and *L. muta* venoms. Furthermore, previous analysis of structure-activity relationship of derivatives has already performed [25]. The derivatives were submitted to the analysis of “Lipinski's rule of five” that indicates that a chemical molecule could be an orally active drug in humans and such a rule states that a molecule violating any two of the following rules is likely to be poorly absorbed: (1) molecular weight less than 500 Da, (2) number of hydrogen bond donors (OH or NH groups) equal or less than 5, (3) number of hydrogen bond acceptors less than 10, and finally (4) calculated *c*Log *P* less than 5 [41]. The results showed that all derivatives fulfilled this rule (molecular weight = 296.31–341.30; *c*Log *P* = 2.6–3.4; *n*HBA = 8–11 and *n*HBD = 1–3) pointing for good theoretical biodisponibility [25].
